# Treatment gaps in epilepsy

**DOI:** 10.3389/fepid.2022.976039

**Published:** 2022-08-01

**Authors:** Jacob Pellinen

**Affiliations:** Department of Neurology, University of Colorado School of Medicine, Aurora, CO, United States

**Keywords:** neurology, diagnoisis, underutilization, healthcare delivery, disparities, accessibility, barriers

## Abstract

Over 50 million people around the world have epilepsy, and yet, epilepsy recognition and access to care are ongoing issues. Nearly 80% of people with epilepsy live in low-and middle-income countries and face the greatest barriers to quality care. However, there are substantial disparities in care within different communities in high-income countries as well. Across the world, under-recognition of seizures continues to be an issue, leading to diagnostic and treatment delays. This stems from issues surrounding stigma, public education, basic access to care, as well as healthcare worker education. In different regions, people may face language barriers, economic barriers, and technological barriers to timely diagnosis and treatment. Even once diagnosed, people with epilepsy often face gaps in optimal seizure control with the use of antiseizure medications. Additionally, nearly one-third of people with epilepsy may be candidates for epilepsy surgery, and many either do not have access to surgical centers or are not referred for surgical evaluation. Even those who do often experience delays in care. The purpose of this review is to highlight barriers to care for people with epilepsy, including issues surrounding seizure recognition, diagnosis of epilepsy, and the initiation and optimization of treatment.

## Introduction

Decades of work have gone into identifying and bringing to light treatment gaps in epilepsy. Despite this work, progress to significantly narrow care divides has been slow. Over the years, issues related to education, stigma, and treatment delays have been consistently reported throughout different regions. At the same time, there have been efforts to improve epilepsy care, particularly in low- and middle-income countries where the treatment gap is greatest. However, significant barriers remain. Even people in high-income countries continue to experience barriers to care such as a lack of specialists, underutilization of epilepsy surgery, and variable resource allocation (1).

**Figure 1:**
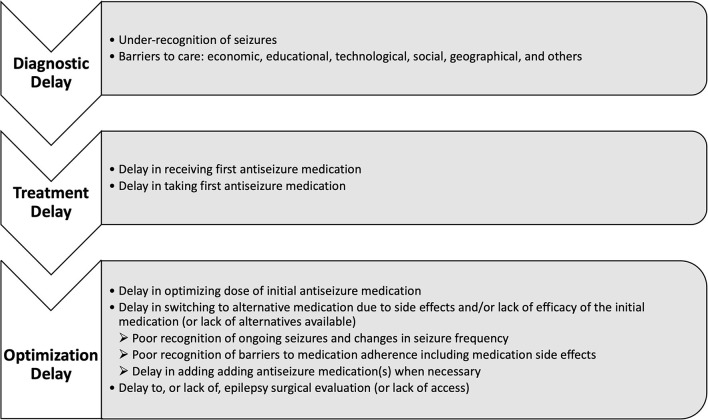
Conceptual framework for the timeline of diagnostic delay in epilepsy and specific barriers at each stage.

Gaps in epilepsy care range from lack of access to care and delayed diagnosis, to delayed treatment and lack of treatment optimization (Figure 1). Recently, this was synthesized in a systematic review aimed at standardizing the definition of the treatment gap and broadly including key drivers into two new primary definitions. First, a conceptual definition, which refers to the overall proportion of people with active epilepsy who do not receive appropriate treatment, and second, an operational definition, which refers specifically to the difference between the total number of people with active epilepsy and the number of those whose seizures are being appropriately treated (2). Standardization of the definition is important for improving the quality of reporting, and enabling higher quality meta-analysis in this area of research. Beyond quantifying the treatment gap, identifying barriers to care, and improving the quality of reporting, collaborative efforts in clinical care, research, education, and advocacy are critical for developing sustainable improvements (3). This review discusses well-recognized gaps in epilepsy care from across the spectrum of barriers to treatment optimization, as well as highlighting ongoing efforts in improvement.

## Seizure recognition

Difficulties recognizing seizures by patients, families, and healthcare workers is a prominent issue often leading to delays in diagnosis and treatment. In general, the types of seizures least well-recognized are those without motor manifestations. These non-motor seizures can be outwardly subtle and go unassessed for long periods. Over time, recurrent non-motor seizures often culminate in bilateral tonic clonic convulsions, which ultimately prompt medical evaluation and most often lead to a diagnosis of epilepsy.

Recently, data from the Human Epilepsy Project (HEP) has shown substantial delays to diagnosis for people with focal epilepsy. Most striking, the delay to diagnosis was 10-times longer in people who initially experienced only non-motor seizures compared to those with motor seizures at epilepsy onset (4). This adds to evidence from several studies over the past decade that have reported substantial delays in diagnosis for people with epilepsy, primarily those with focal epilepsy, and particularly in those with epilepsy characterized by non-motor seizures at epilepsy onset (5, 6). In addition to poor recognition of seizures, a recent review on the topic clarified three primary drivers for the diagnostic delay: “decision delay” (patient-deferred evaluation), “referral delay” (lack of specialist referral), and “attendance delay” (lack or delay of evaluation when referred) (7). There is also evidence that people with new-onset epilepsy related to high-grade tumors, strokes, and older age tend to have a faster times-to-diagnosis than others, highlighting that delay may also vary depending on etiology and age (8). Areas for potentially improving seizure recognition and narrowing these gaps in care will be discussed in regard to specific barriers in the following sections.

### Public education and social stigma

Ongoing social stigma surrounding the diagnosis of epilepsy can lead to delayed diagnosis, preventing people from seeking timely medical evaluation. One way to reduce stigma is to improve the public understanding of seizures and epilepsy through education, which can also lead to improvements in diagnosis and treatment. The development of public health campaigns for many diseases have shown success. Within neurology, this has been particularly successful for stroke treatment, in which public awareness campaigns, mnemonics, and quality improvement have dramatically improved the quality of care (9). In designing public awareness campaigns, it is important to understand baseline public knowledge on the topic through utilization of qualitative methods so that such efforts can be aimed at improving true gaps in awareness and knowledge (10, 11). To date, this represents a relative shortcoming in the literature, as baseline public knowledge has been largely investigated through survey studies in different regions around the world rather than using qualitative methods. Although large surveys can have issues from variability in comprehension of questions to the environmental context in which they are asked, these have nonetheless shed light on important themes. For instance, these have shown that although the stigma surrounding epilepsy varies by country and region, it persists in all regions regardless of resources, education, and income level. Unfortunately, door-to-door surveys are problematic due to the social stigma surrounding epilepsy in many of the locations where they are carried out. Specifically, people with epilepsy who have infrequent seizures, or are in remission, may be less likely to disclose their medical history due to the fear of stigmatization with no immediate benefits from responding to surveys accurately (12).

In Africa, notable surveys on the topic include a one conducted in Rwanda in 2005, in which over a thousand individuals were sampled *via* random cluster sampling, and found that most respondents believed people with epilepsy should not be allowed to go to school (66%), to work (72%), to use public spaces (69%), or to get married (66%), and believed that epilepsy was untreatable (50%) and transmissible (40%) (13). An even larger door-to-door survey including 4500 people in suburban Senegal the same year found that 51% of respondents believed epilepsy was caused by evil spirits and 35% believed it to be contagious (14). The treatment gap in Tanzania as recently as 2016 was reported to be ~45% and had the highest correlations with a lack of education or knowledge of epilepsy or believing in a supernatural cause for epilepsy (15). Even in high income countries, there are gaps in knowledge among people with epilepsy regarding their diagnosis as well as high rates of perceived stigma (16). Stigma exists to variable degrees in all environments, and takes different forms based on local cultures, medical traditions, economic conditions, and politics (17). It can also be substantial among immigrants, which was recently reported in a study carried out in Sweden, where feelings of social isolation were magnified in people with epilepsy who were facing language barriers and an unfamiliar healthcare system (18). These examples underscore the common themes that have been present for thousands of years, and have led to discrimination against people with epilepsy that persists to this day (19).

Current evidence points to an ongoing poor public understanding of seizures and epilepsy. This can be improved through educational initiatives for people with epilepsy, their families, and their communities, and may be a fundamental for improving stigma. An important finding from efforts to identify and reduce stigma has been that public awareness and epilepsy education is negatively correlated with stigma (20, 21). This was confirmed in a recent study in Pakistan, where efforts to improve public awareness of epilepsy led to a significant reduction in both the epilepsy treatment gap as well as stigma (22). These studies highlight a clear target for improving the quality of life and quality of care for people with epilepsy.

### Socioeconomic and technological divides

Socioeconomic disparities, as well as frequently co-occurring technological divides, underlie the most significant gaps in epilepsy care worldwide. In several regions, there are ongoing issues with people not being able to access and afford an antiseizure medication, let alone having more than one medication option (23). Beyond basic access to medication, there are also stark differences in quality of life and quality of care for people depending on demographic variables. For many years there has been an ongoing collaborative effort to steer epilepsy research in the direction of identifying and addressing such disparities in care by leaders in the field (24).

Several studies in the United States and North America have shown different rates of seizure control and epilepsy remission along racial and ethnic divides, including delays to diagnosis and decreased utilization of epilepsy surgery in several minority populations (25–27). There have been several studies from different regions around the world highlighting significant differences in quality of life and quality of care along socioeconomic and racial divides (28–31). A recent investigation has also shown higher rates of sudden unexpected death in epilepsy (SUDEP) in people with high socioeconomic disadvantage, which persisted during the study for those with the highest socioeconomic disadvantage over a time when overall rates of SUDEP were decreasing (32). Many barriers to care that have been consistently identified over the years have clear potential for intervention, such as improving medication affordability and improving access to primary and specialist care. However, these issues frequently necessitate larger political solutions as well as healthcare systems solutions and often face economic and political barriers. Strategies to improve access to care are expanding significantly as communication technology improves and telehealth services expand. However, sustainable solutions will likely still rely on a coordinated effort between individuals and communities along with healthcare institutions, government, and non-governmental organizations.

### Healthcare worker education

The most widely reported factor contributing to diagnostic delay is under recognition of common seizure symptoms. Specifically, seizures with outwardly subtle symptoms, such as non-motor seizures, are under-recognized (33). Not only are patients more likely to seek medical evaluation after experiencing convulsions, but healthcare workers are more likely to recognize and treat them. A large retrospective cohort study in the United States showed that approximately one-third of patients with newly diagnosed epilepsy remain untreated up to 3 years following an initial diagnosis (34). This study also highlighted the fact that variability of seizure symptoms at epilepsy onset can present a lengthy differential on initial evaluation, which inevitably leads to treatment delays (35). Even when patients present to emergency departments for evaluation of convulsive seizures, a history of preceding seizures (often non-motor) is present in up to half and go largely unrecognized or untreated (36–40). This is a large group of patients who are seen by healthcare providers at a time when they meet diagnostic criteria, yet remain undiagnosed. Additionally, healthcare providers often also fail to adequately identify important family history during initial evaluations. This is an important factor not only for diagnosis, but can have implications for prognosis and counseling, such as in the case of familial mesial temporal lobe epilepsy, which often is not recognized due to not obtaining an adequate family history (41). It is possible that improving seizure education for healthcare workers may improve the quality of medical evaluations when patients present with seizure symptoms and may help to narrow this gap in care.

A study in Nepal found that it is possible to train non-neurologists to accurately diagnose epilepsy in resource-limited settings (42). This is a reasonable goal for making improvements, particularly in low- and middle-income countries where there can be a significant knowledge gap about epilepsy among primary healthcare workers (43). However, even non-neurologist healthcare workers are limited in many low-income countries, and so mindfully distributing primary medical care resources is important for making sustainable improvements (44). A growing opportunity to improve education and awareness among healthcare workers in all settings is technology – web seminars, pre-recorded lectures, and other online learning can supplement training and may be a way of improving epilepsy education particularly outside of large academic centers where people have access to technology (45).

## Gaps in treatment

In response to recognition of the pervasive treatment gap in epilepsy, the International League Against Epilepsy (ILAE), International Bureau for Epilepsy (IBE), and World Health Organization (WHO) started the Global Campaign against Epilepsy in 1997. Shortly thereafter, the National Institute of Neurological Disorders and Stroke (NINDS) in the United States in collaboration with the American Epilepsy Society (AES) established benchmarks for epilepsy research in 2001 in order to guide future progress (46). In 2006, results from the collaborative ILAE/IBE/WHO Global Campaign Against Epilepsy, in which data were collected from 160 countries, confirmed a significant gap in epilepsy treatment in the majority of countries with substantial regional variability resulting from economic disparities (47). Subsequently, there were additional reports increasing epilepsy awareness, including a 2015 resolution by the World Health Assembly (WHA) urging member states to implement a coordinated action against epilepsy and its consequences (48). Then in 2020, the 73^rd^ WHA unanimously approved a resolution to develop and implement a 10-year global action plan on epilepsy, and after being discussed by the Executive Board was recommended to be adopted by the 75^th^ WHA in May 2022 (49, 50).

In examining gaps in treatment, a common issue with systematic reviews on the topic is the tendency to exclude non-English publications and exclude many studies on populations in low-income countries, which can lead to sampling bias (51). There is also variation in the definition used for treatment gap, and substantial variability in quality and reporting, making quality meta-analysis challenging. In much of the literature, the epilepsy treatment gap has referred to the proportion of people with untreated epilepsy relative to the prevalence of active disease (52). This is most similar to a recently proposed conceptual definition, though the newer definitions take into account many factors that are important to this issue including gaps in affordability of care and medications, diagnostic gaps, therapeutic gaps, and other issues related to quality of care (2). This effort to standardize the definition is important for ongoing efforts to improve the quality and transparency of reporting, and the consistency between studies. Taking current studies into consideration, gaps in epilepsy care range from poor utilization of antiseizure medications, to poor optimization of antiseizure medications, and poor utilization of epilepsy surgery.

### Gaps in antiseizure medication use

A consistent finding across regions is that the most vulnerable people experience the highest treatment gap. Worldwide disparities in care are most striking between areas of high and low socioeconomic status, with high-income and urban areas having the lowest treatment gaps and low-income and rural areas having the highest treatment gaps (2, 53, 54). Since the primary determinant of the treatment gap worldwide appears to be directly related to economic and healthcare-related resources, it is largely a reflection of basic access to medication and healthcare services (55).

Reviews of the treatment gap in different regions have highlighted this point powerfully. In Africa, a recent review of the epilepsy treatment gap among sub-Saharan countries showed a collective treatment gap from 23 studies of nearly 70% (56). A door-to-door questionnaire study conducted in Egypt on 33,818 people found a treatment gap of 83.8% (57). In some African countries the treatment gap has been reported to be particularly high, such as in Madagascar, where an estimated 92% of people with epilepsy remain untreated (58). On other continents, the treatment gap is likewise particularly significant in rural and low-income communities. A door-to-door survey conducted on 55,000 people in China found that 41% of people with epilepsy had never received appropriate treatment and 63% with active epilepsy had not been taking antiseizure medication in the week prior to the survey (59). A separate cross-sectional analysis of 54,976 people in Eastern China found a treatment gap of 58.5%, which was independently associated with having a high seizure frequency and not having health insurance (60). Similar findings have been seen in other studies across Asia from Vietnam and the Philippines to South Kazakhstan, where the treatment gap ranges from 25% to nearly 85%, and has been attributed to socioeconomic barriers, poor public education, limited access to care, limited access to medications, suboptimal use of medications, and unaffordability of medications (61–63). One study from South America found that only about 50% of people with epilepsy São Paulo, Brazil, were taking an antiseizure medication (64).

These reports all paint a bleak picture of the treatment gap, but an even more unfortunate reality is that despite recognition of the problem, there have been few substantial improvements over time. A large systematic review for the WHO published in 2010 reported a gap of 75% in low-income and 50% in most middle- to upper-income countries, but most striking was the finding that over a 20-year period form 1987 to 2007 there was no improvement in the treatment gap (53). Part of this stems from a lack of seizure recognition, which leads to delayed diagnosis on the front end, as well as underutilization of antiseizure medications in people with known diagnoses. This is compounded by issues discussed earlier related to context and location – stigma, education, and socioeconomic variables including access to care and technology. One area to improve the treatment gap, however, may be to improve seizure recognition.

Previous studies have found that when people are evaluated in emergency settings for convulsions, a diagnosis of epilepsy is often missed due to lack of recognizing preceding seizures (5, 36). Improving assessments for people seeking emergency care for first time seizures may have a meaningful impact on improving the time to diagnosis and treatment. This could either be an improvement within the emergency departments, or creating separate first-seizure clinics, which have been successfully implemented in some centers in Australia and shown to improve time-to-diagnosis and treatment (65). Initial diagnosis and treatment is crucial, but delay in this process is not the only gap in care experienced by people with epilepsy.

Once a diagnosis is made, optimizing treatment to prevent further seizures and minimize medication side effects is also crucial. This is an additional layer of complexity that can vary in magnitude depending on local resources. There is substantial variation in medications prescribed for people with epilepsy based on demographic and socioeconomic factors that are often not in line with current recommendations (66, 67). In other words, there are gaps in treatment optimization and the long-term epilepsy care following treatment initiation. One potential way of improving and standardizing care across health systems has recently been explored among children and youth with epilepsy in Project ECHO (Extension for Community Healthcare Outcomes). This model utilizes a hub-and-spoke knowledge-sharing network to leverage expert knowledge in supporting improved care of specialty conditions being treated by a larger network of primary care providers, and was successful when used for improving the quality of care for children and youth with epilepsy (68). Such solutions will be important for improving quality of care considering the limited numbers of specialty-trained physicians, and are increasingly possible through the expanding use of new technologies.

### Gaps in epilepsy surgery utilization

Many patients with drug-resistant focal epilepsy who undergo epilepsy surgery are substantially more likely to be seizure free following surgery than those who remain on antiseizure medications alone (69, 70). However, the under-utilization of surgical treatment options for drug-resistant epilepsy has been well described over the years despite evidence to support its use. Even with growing evidence to support epilepsy surgery, as well as several calls-to-action, there remains a persistent knowledge gap among both physicians and patients, as well as a lack of federal funding for research in this area compared to other medical specialties (71, 72). There is also a gap between clinician knowledge and their actions – i.e., even if they are educated in regard to surgical treatment options, there is nonetheless an under-utilization of epilepsy surgery that represents a significant treatment gap (73). Furthermore, epilepsy surgery has been shown to be underutilized in a high-income universal health system, suggesting that access to surgical centers is not the only barrier to utilization (74).

Not only is surgery underutilized in high-income countries, but there are disparities among those who receive it based on age, race, and health insurance (75). In low-income countries the issue is more pronounced since there are limited centers capable of performing epilepsy surgeries. Children face substantial barriers to attaining epilepsy surgery even when it is indicated, often the result of poor understanding of surgery on the part of their families and healthcare providers, as well as due to system disparities in care (76). In all settings, there is a combination of patient-related factors, physician-related factors, and health system factors, which factor into underutilization of epilepsy surgery (77). One method for improving this gap in care may be to expand involvement of epilepsy specialists early in the care of people with epilepsy through the creation of educational networks such as how Project ECHO was utilized for improving the care for children and youth with epilepsy as discussed above. By making inroads into primary care practices and general neurology practices, it may be possible to increase the number of referrals to epilepsy surgical centers and increase acceptance of this as a treatment option among patients and non-specialists.

## Conclusions

Despite continual advances in treatment options for epilepsy, there remain significant barriers to care across the world (Table 1). Disparities exist among communities in all countries, with the greatest gaps in care in low- and middle-income countries. Although recognition of epilepsy has been increasing over time, there are still significant barriers to timely diagnosis and treatment due to under-recognition of seizures. These include issues related to stigma, access to care such as economic, technological, and language barriers, as well as under-recognition of seizures and epilepsy among both the public and healthcare workers. Even once diagnosed, people with epilepsy often face gaps in the optimization of seizure control with the use of medications and surgical evaluation when needed.

**Table 1:** Examples of barriers to epilepsy diagnosis and treatment.

**Epilepsy diagnostic status**	**Barriers**
Pre-diagnosis	• Distance to healthcare facility • Local customs or beliefs • Lack of seizure unknowledge • Superstitions • Discrimination and stigma • Lack of family/social support • Denial or minimization of symptoms • Lack of diagnostic services (e.g., EEG) • Poor healthcare worker education
Post-diagnosis	• Cost and availability of medications • Side effects of medications • Inefficacy of medications • Cost of medications • Medication non-compliance • Drug resistance • Distance to healthcare facility • Cost of medical care • Lack of specialists • Lack of seizure/epilepsy education • Lack of caregiver support • Discrimination and stigma • Medical comorbidities

## Author contributions

JP was solely responsible for concept, design, and drafting of the manuscript.

## Conflict of interest

The author declares that the research was conducted in the absence of any commercial or financial relationships that could be construed as a potential conflict of interest.

## Publisher's note

All claims expressed in this article are solely those of the authors and do not necessarily represent those of their affiliated organizations, or those of the publisher, the editors and the reviewers. Any product that may be evaluated in this article, or claim that may be made by its manufacturer, is not guaranteed or endorsed by the publisher.
